# Brophy’s Oral Surgery

**Published:** 1915-12

**Authors:** 


### BIBLIOGRAPHY

BROPHY'S ORAL SURGERY. The long-expected work on Oral Surgery by Prof. Truman W. Brophy has just issued from the well-known medical publishing house of P. Blakiston's Son & Co. It is a large volume of over 1,000 pages, with over 900 illustrations, many of which are colored. It is difficult to estimate the comparative values of books even on the same subject and we find it especially difficult to compare this book with any other similar work, for the reason that this book is the individual record of a life devoted to the subject by one who did his work to so large an extent from the viewpoint of the dental teacher and practitioner. We are pleased to find that the book is very largely a compilation of the results of a long life of research and experience and is neither narrow in conception nor pedantic in expression. In reading the book we are constantly reminded of Garretson, who was a master of expression because his style was pure and graphically expressive. The book will appeal to every one mostly because it is the record of an intense devotion to an effort to establish the science and art of Oral Surgery as an important element of the dentist's equipment. Dr. Brophy is a dentist first and a medical scientist perforce, but his attitude of mind is that of the dental practitioner. His work therefore will appeal to the profession with more force than had he written from the general surgeon's viewpoint, or even as a dentist who has taken his cue from the standard medical works.

The work is too large for us to make a critical review even had we the ability. We have examined the work sufficiently to see that it is probably the best work on this subject, certainly it will meet the requirements of the profession of dentistry more adequately than any other book that has been printed on this subject. It is so comprehensive that it covers every department of dental surgery, and while undoubtedly some may differ in the technical treatments, the fundamentals of practice are clearly and systematically carried out in every chapter. It is in a way a cyclopedia of information, and will become our standard as a work of reference. No dental library of any pretensions can be without it—in fact it should be placed in the hands of every practitioner. The author has perhaps rendered to his beloved profession his greatest service and we not only congratulate him on the excellence of his endeavor, but trust he may live many years to enjoy the larger sphere of influence which this book will give him. Some books are conceived and produced for the purpose of personal aggrandizement and we trust Professor Brophy may realize some of these very proper benefits, but this book does not seem to have had, nor in fact does it need, any of these ulterior objectives, to give a reason for its existence. It is the normal and irresistible consequence of a life spent in helping to perfect the art and science of Oral Surgery. As was to be expected the book contains a complete disclosure of the Brophy methods of treating Hare-lip and Cleft Palate operations, which while not of so much interest to the general practitioner as is most of the other matters treated in the work, will put on record for all time the most perfect technical treatment of these most offensive deformities. The book is too large and expensive to become a generally used text-book for student use, but it undoubtedly will find a prominent place in teaching fields where all knowledge must be placed before the students. No practitioner can afford to be without this work in his library, as it treats scientifically every oral disease from diagnosis to resolution.

